# A novel hollow-fiber infection model (HFIM) for antiviral PK/PD studies of CMV infection

**DOI:** 10.1128/aac.00381-26

**Published:** 2026-06-18

**Authors:** Lalitya M. Sudarsono, Suzanne A. M. Wenker, Xuanlin Liu, Jorn Brink, Dirk-Jan van den Berg, J. G. C. van Hasselt, Anne-Grete Märtson

**Affiliations:** 1 Division of Systems Pharmacology and Pharmacy, Leiden Academic Centre for Drug Research, Leiden University 4496, Leiden, Netherlands; Providence Portland Medical Center, Portland, Oregon, USA

**Keywords:** CMV, antiviral agents, PK/PD, ganciclovir, hollow fiber infection model, cytomegalovirus

## Abstract

The hollow-fiber infection model (HFIM) is a translational *in vitro* model that links time-varying human pharmacokinetic profiles to the associated viral dynamic responses, from which pharmacokinetic/pharmacodynamic (PK/PD) targets can be derived. Establishing such targets is essential for antiviral dose selection and optimization. This is particularly important for cytomegalovirus (CMV) infection treatment, which primarily affects vulnerable patient populations. PK/PD targets for ganciclovir, the first-line drug for treatment, have not yet been defined. The lack of defined targets makes dose optimization challenging and may result in suboptimal exposure, prolonged toxicity, and the emergence of resistance. For the first time, we have demonstrated the use of a low-cost hemodialyzer hollow-fiber cartridge for CMV infection and ganciclovir treatment studies. We have established a system that i) supports CMV culture for PD analysis, ii) reproduces a clinically relevant ganciclovir PK profile, and iii) maintains consistent drug exposure in the infected cells, allowing reliable PK/PD analysis. Quantitative methods such as tissue culture infectious dose 50% (TCID_50_) and quantitative PCR were used to assess both viral titer and viral genome production. Ganciclovir PK was measured using liquid chromatography-tandem mass spectrometry (LC-MS/MS). This validation study serves as a fundamental step that can allow further PK/PD studies for ganciclovir and other antiviral agents that remain largely understudied. Consequently, this model could provide an affordable and practical platform for establishing clinically relevant PK/PD targets and guide treatment optimization.

## INTRODUCTION

Pharmacokinetics/pharmacodynamics (PK/PD) play a central role in anti-infective drug development and treatment optimization. For antivirals, PK/PD is essential for understanding how, for a given compound, the exposure dynamics over time can be linked to its viral dynamic outcomes. In antimicrobial therapy, the PK/PD for several antibiotics and antifungals such as glycopeptides, aminoglycosides, and triazoles is well defined. This understanding refers to whether a compound is concentration-dependent, time-dependent, and/or both concentration- and time-dependent to achieve an optimal therapeutic target. It is, however, still unclear whether the same principles apply to antivirals as the exposure-response (PK/PD) targets have not yet been well characterized nor established (1).

The HFIM is an *in vitro* system that is primarily used to reproduce human PK and evaluate the expected PD response. While commonly used for antibiotic studies, the use of the HFIM is less common in antiviral studies (2). Mimicking the human PK is critical as drug clearance and exposure over time can have a significant influence on viral load decline (1, 3, 4). These variables cannot be mimicked in conventional *in vitro* assays, where static concentrations are maintained over time and clinical dosing regimens cannot be tested (5–7). Currently, there are limited PK/PD studies available for antivirals. This is particularly evident in the case of cytomegalovirus (CMV) infection treatment, where PK/PD targets have not been well established (8, 9).

CMV is a latent herpesvirus that causes severe diseases and high mortality rates in immunocompromised individuals, which are often reported in transplant recipients (10). Ganciclovir and its prodrug, valganciclovir, are the first-line drugs of choice for CMV treatment in clinical practice (11, 12). However, treatment with ganciclovir is challenging due to undefined therapeutic targets and high frequency of clinical toxicity (8, 13, 14). Limited PK/PD analyses have been done for alternative anti-CMV drugs such as foscarnet, where PK/PD targets are yet to be defined (15). Given the limited PK/PD understanding of current anti-CMV therapies, there is an urgent need to define such targets for achieving better clinical outcomes in CMV treatment (14–18).

In this study, we aimed to establish and characterize a robust HFIM that can support CMV infection while reproducing a human-like PK profile of ganciclovir. Our main objective was to provide a reproducible and affordable framework for PK/PD assessments of ganciclovir, which can be extended for other antiviral agents, with the use of a low-cost hemodialyzer.

## RESULTS

### CMV replicates actively in the established HFIM

**Fig 1:**
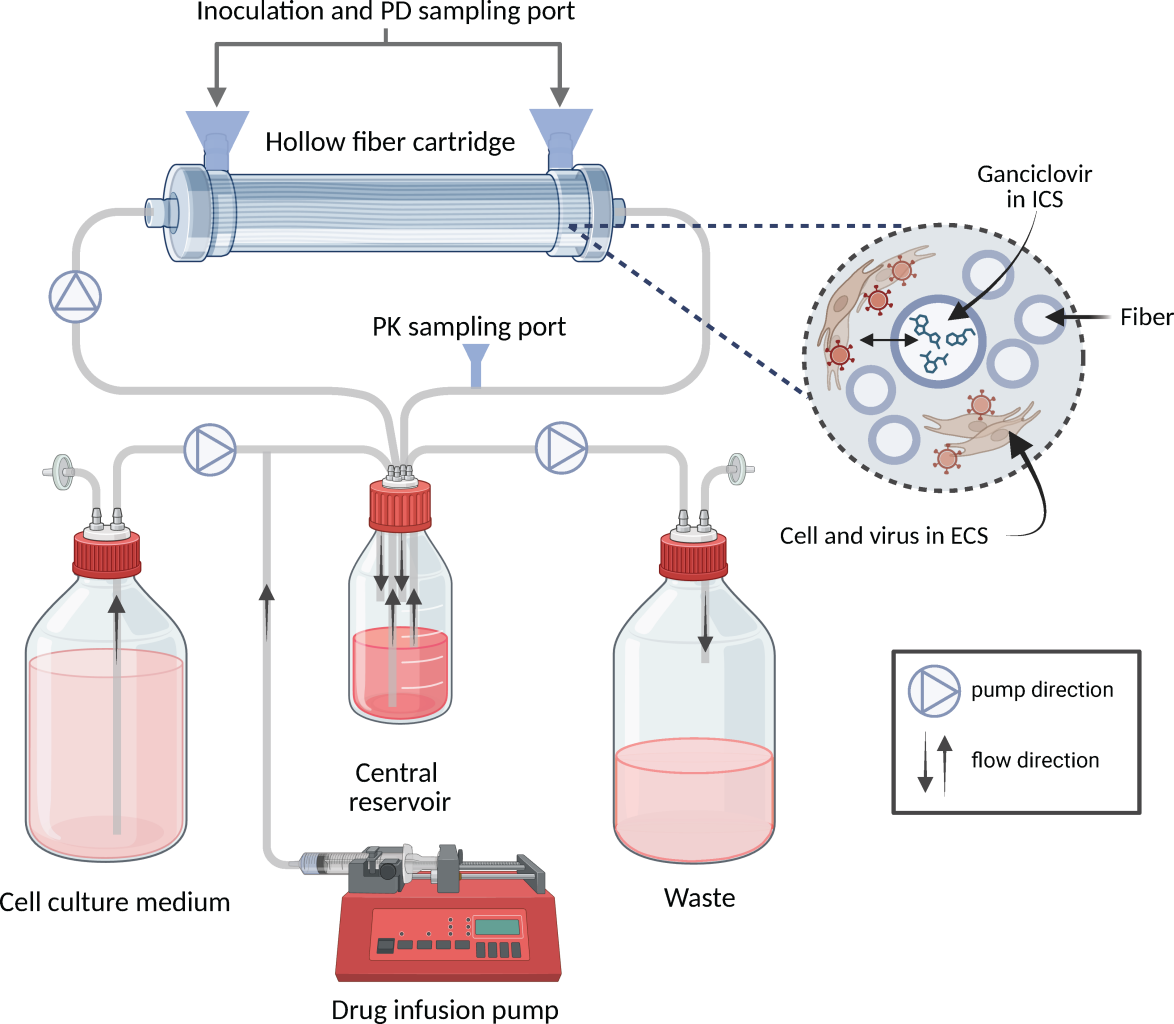
Schematic overview of the hollow-fiber infection model. MRC-5 cells and CMV were inoculated into the extra-capillary space (ECS) of the hemodialyzer cartridge. A continuous flow of the cell culture growth medium into the cartridge was maintained from the central reservoir. For the drug-treated system, ganciclovir was infused directly into the central reservoir and flowed along the intracapillary (ICS) of the hollow-fiber. The hollow-fiber allows ganciclovir to diffuse between the ICS and the ECS, while cells and virus are retained in the ECS. To mimic drug clearance, an inflow of the drug-free, cell culture medium was pumped into the central reservoir, and the excess volume was flowed out into the waste. The cartridge and central reservoir were maintained at 37˚C and 5% CO_2_ during the experiment.

**Fig 2:**
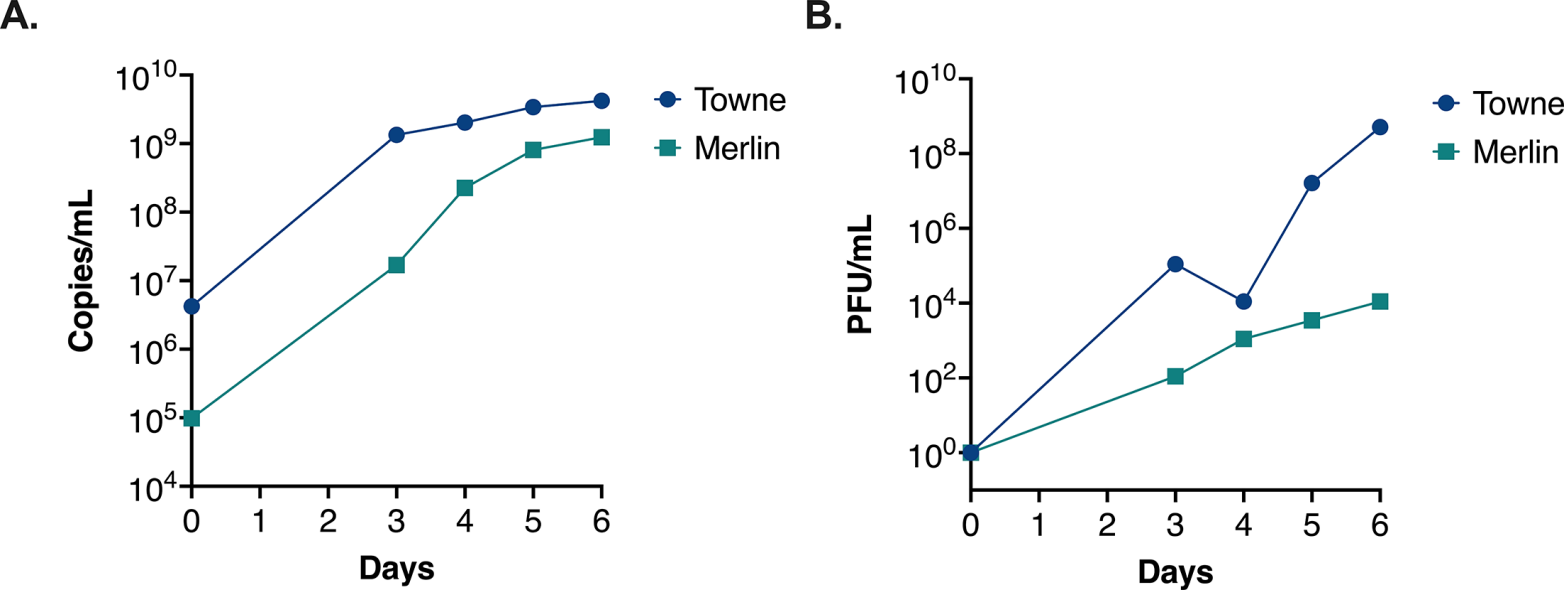
CMV replicates in MRC-5 cells within the HFIM system. Circle and square points represent the Towne (lab-adapted) and the Merlin (wild-type) strain, respectively. **A** and **B** represent the genome copies (copies/mL) and viral titer (PFU/mL), over 6 days of infection period at 37˚C/5% CO_2_, respectively.

We established the hollow fiber-CMV infection setup, as schematically represented in Fig. 1; further details on culturing conditions and the framework of the setup are described in Methods. The hollow-fiber cartridge is used to support CMV infection, where a continuous flow of the cell culture growth medium from the central reservoir is maintained throughout the cartridge. It serves as an infection site, where the human lung fibroblasts (MRC-5) cells and CMV strains were inoculated and retained. To evaluate whether our system can support CMV infection, we used two strains: the Towne, a lab-adapted strain, and the BAC-derived, Merlin strain, which is genetically similar to the wild-type strain (19). An increase in both genome and viral titer production was achieved throughout 6 days of the infection period (Fig. 2). Consequently, we observed a 3-and 4-fold log_10_ increase in genome accumulation (copies/mL) for Towne and Merlin strains, respectively. Meanwhile, a 4- and 10-fold log_10_ replication of active viral titers (PFU/mL) were observed for the Merlin and Towne strains, respectively, over 6 days of the infection period (Fig. S1). The system could, therefore, support a long-term infection period, which can be used for further PD assessments.

### HFIM reproduces human-like ganciclovir PK parameters and plasma profile

**Fig 3:**
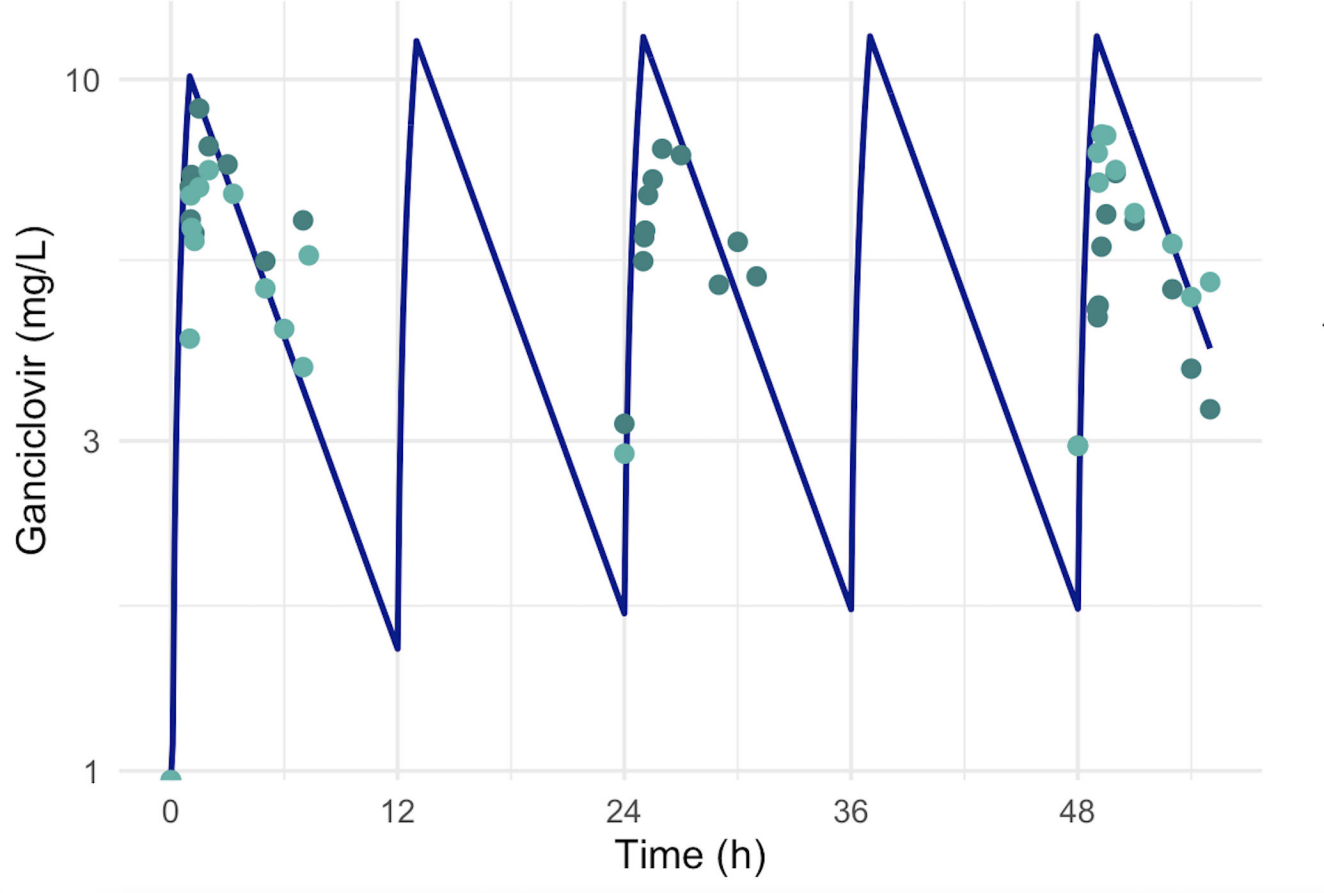
Concentration-time profile of observed (points) and simulated (line) ganciclovir concentrations. Blue and teal circles represent data points from two independent experiments.

Ganciclovir was infused directly into the central reservoir, as represented in Fig. 1, at 2 mg/h (1-hour continuous infusion), administered every 12 hours (q12H), as in clinical practice. To evaluate whether the HFIM can reproduce a human-like PK, we simulated expected PK curves based on the input parameters. The observed and the simulated concentration-time profile were compared, and similar profiles were achieved (Fig. 3). The observed data were fitted to a one-compartmental model. Thereby, the clearance, volume of distribution, elimination constant rate (K_e_), and 12-h area under the concentration-time curve (AUC_0-12h_) were also estimated using a one-compartmental model fitting (Table 1). We have observed an overall good fit of the observed PK profiles. Therefore, clinical dosing regimens of ganciclovir can be performed in the established HFIM.

**TABLE 1:** Input vs estimated ganciclovir PK parameters of the HFIM with one-compartmental fitting analyzed in R

PK parameters	Input	Estimates (%RSE)
Clearance (L/h)	0.03	0.033 (0.2)
Volume of distribution (L)	0.2	0.3 (2.05)
Elimination rate constant (h^−1^)	0.15	0.11
AUC_0-12_ (mg.h/L)		42.2

### Ganciclovir maintains active exposure into the infection site without binding in the HFIM system

**Fig 4:**
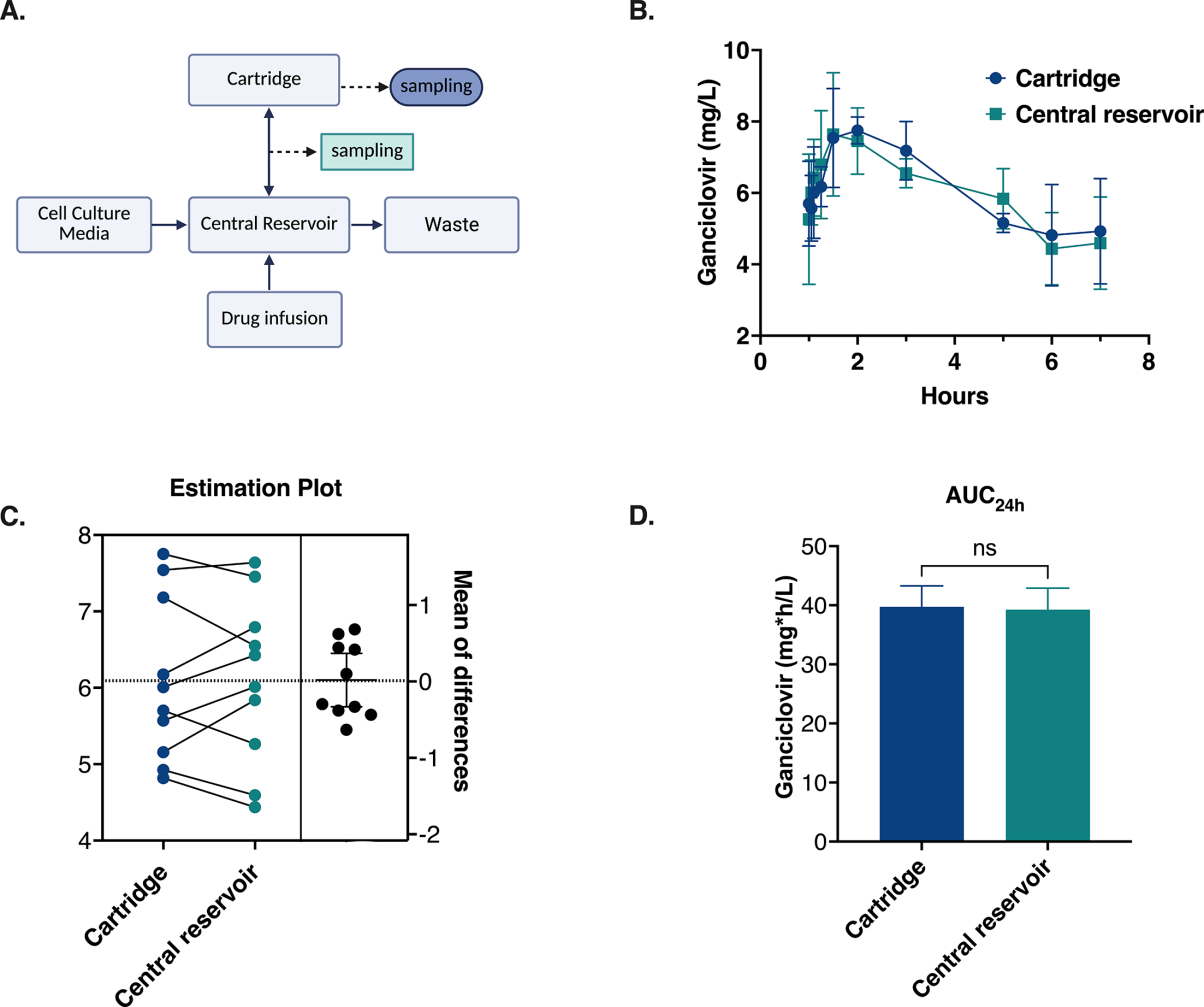
Ganciclovir concentrations in the hollow-fiber cartridge (ICS) and in the central reservoir (ECS) were compared. (**A**) Schematic of the HFIM system showing the two sampling ports. (**B**) Ganciclovir concentration profile. (**C**) Differences and mean of differences in concentrations between the cartridge and central reservoir sampled at different time points. (**D**) AUC_24h_ of ganciclovir in the cartridge and central reservoir of the HFIM (*P*-value > 0.05, *N* = 3).

A high flow rate (30 mL/min) was set up to achieve a constant equilibrium between ganciclovir concentrations in the central reservoir, the extra-capillary space (ECS), and the intra-capillary space (ICS) of the hollow-fiber cartridge. To validate whether this is consistent over time, we sampled media containing the drug from the ICS, which comes directly from the central reservoir, and from the ECS, where the cells and virus are retained, at various time points (Fig. 4A). We have observed that the ganciclovir PK curve of the cartridge is similar to that of the central reservoir (Fig. 4B.) Throughout different sampling time points, we observed no significant difference between ganciclovir concentrations as shown by the estimation plot (Fig. 4C). Additionally, no significant differences were observed for the calculated AUC_24h_ between the two sampling points (Fig. 4D). These results demonstrate that the system supported an active diffusion between the ICS and the ECS, while maintaining constant equilibrium between the concentrations in the central reservoir and those to which the cells and viruses were exposed. This also suggests that the compound did not accumulate in the cartridge and no drug binding was present in the system, allowing direct exposure to the infected cells. Therefore, indicating that the time-varying drug exposure within the infection site can be used directly to evaluate the eventual PD response.

### Pharmacodynamics of CMV during ganciclovir exposure

**Fig 5:**
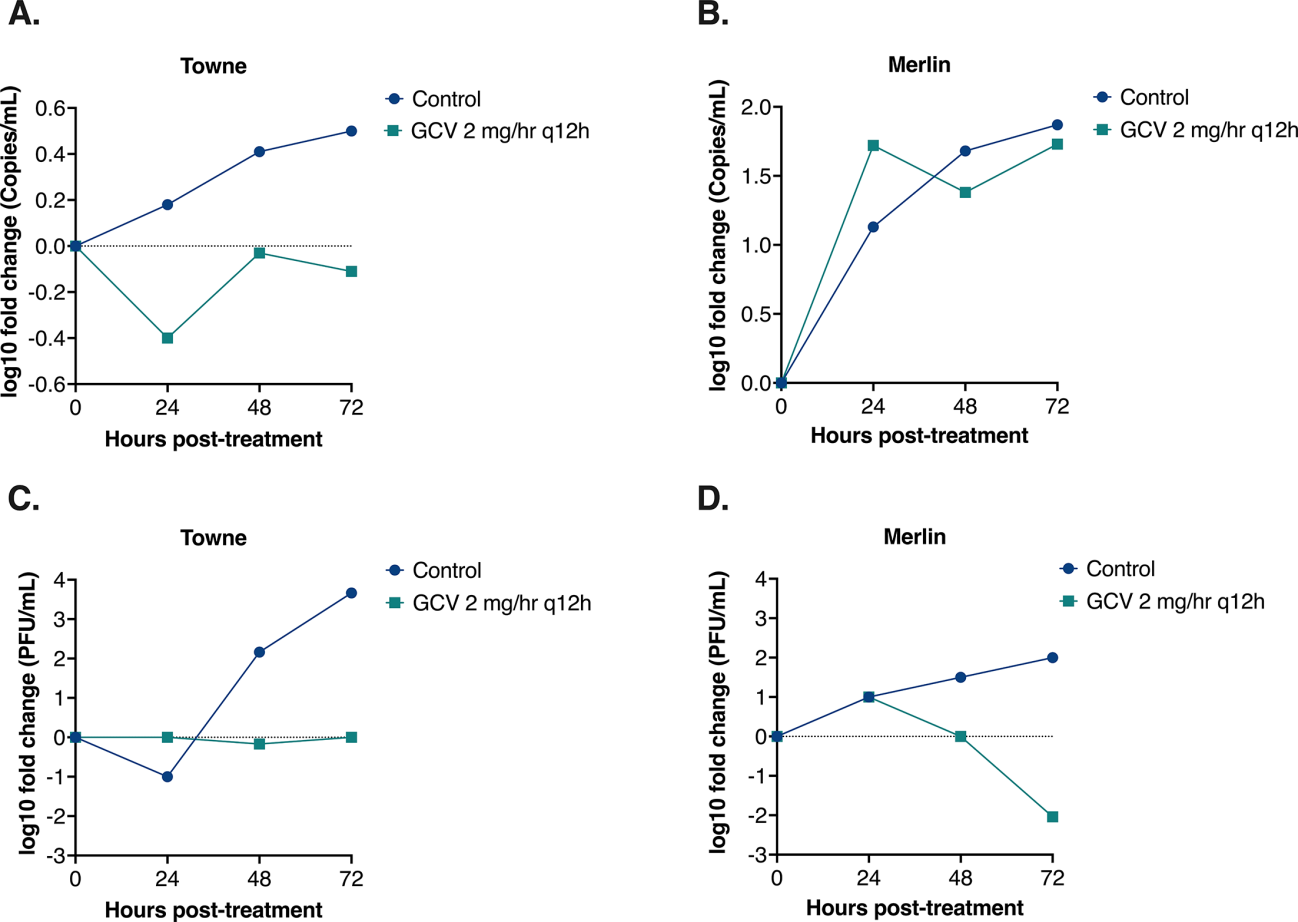
CMV replication with and without ganciclovir treatment in the HFIM. Ganciclovir treatment was administered at 2 mg/hour, as a 1-hour continuous infusion, every 12 hours, for 3 days. Circle and square points represent the control (no drug treatment) and drug treatment group, respectively. (**A–D**) represent the log10-fold change of genome copies (copies/mL) and viral titer (PFU/mL) for Towne and Merlin, respectively, over 72 hours post-treatment initiation.

To characterize the PD response under ganciclovir exposure, we administered ganciclovir after 3 days of viral replication. We then monitored viral replication of both Towne and Merlin strains in terms of DNA copies and active viral titer for 72 hours post-treatment (Fig. S2). To evaluate pharmacodynamic effects, we assessed the log_10_ fold difference relative to the copies or titer observed at day 0, when treatment was initiated. For Towne, we have observed full absence of viral replication for both total DNA copies/mL and viral titer throughout 72 hours of ganciclovir exposure, when compared to the control arm (Fig. 5A and 5C). For Merlin, we initially observed an increase for both genome copies and viral titer after 24 hours post-treatment; thereafter, absence of replication was observed (Fig. 5B and 5D). Upon ganciclovir administration at 2 mg (1-hour continuous infusion, q12H), where AUC_0-12_ at 42.2 mg.h/L was obtained, we observed an overall absence of replication in genome copies and viral titer throughout 3 days post-treatment initiation.

## DISCUSSION

Here, we present for the first time an established low-cost hollow-fiber system that can be applied for ganciclovir treatment optimization studies. Our setup can support a long-term CMV *in vitro* culture for both lab-adapted and wild-type strains, which can allow pharmacodynamic evaluations. Therefore, the system might also be used to amplify different CMV strains in a large volume, which might be useful for infection studies. Importantly, we have reproduced a concentration-time profile for ganciclovir that mimics the human plasma concentration profile with human-like PK parameters. We have also obtained an estimated AUC_0-12h_ that is within the expected clinical target exposure range (40–60 mg.h/L) (20, 21). The result as well confirmed direct ganciclovir exposure to CMV-targeted infected cells within the hollow-fiber cartridge. This means that there was no delay in drug diffusion and distribution between the intracapillary space (ICS) and the extracapillary space (ECS). Consequently, we have observed virus growth suppression when a clinical-based dosing regimen was applied over 3 days post-ganciclovir treatment initiation. Altogether, these results have demonstrated the practicality of the setup for further PK/PD assessments of ganciclovir in CMV infection therapy.

An important advantage of our HFIM system is the use of a hemodialyzer as a low-cost hollow fiber cartridge (up to 25€). The commonly used commercial cartridges are known to be costly (up to 1,000€), which can limit accessibility for academic and high throughput research. The costs can increase exponentially for cell culture, wherein a single-use cartridge is often used for every experimental arm. While recent studies have demonstrated the feasibility of hemodialyzers as cost-effective hollow-fiber cartridges for antibiotic studies (22–25), their application in antiviral studies has not been reported previously. For antiviral studies, the experimental condition is complex as both the host cell and virus culture need to be maintained inside the cartridge. Previous studies have only demonstrated the use of a commercialized HF cartridge for antiviral PK/PD studies. Here, we demonstrate for the first time the feasibility of a hemodialyzer for sustaining viral culture in a HFIM setting, suggesting potential applicability for PK/PD studies of other antiviral agents. Nevertheless, the culturing conditions in the cartridge, such as the cell line, cell number, virus strain, and multiplicity of infection (MOI), might require further optimization. Lastly, it is important to take note that ganciclovir has negligible protein binding, for which the system can disregard. In cases where protein binding is significantly higher, the effect should be considered when establishing a HF setup for PK/PD analysis.

The use of an infection model with a dynamic drug exposure setup is critical for establishing clinically relevant therapeutic targets. Static drug concentration-based assays are limited by the lack of drug clearance, and thus clinical dosing regimens are difficult to be applied (26–28). The established HFIM in this study provides an *in vitro* setup where these limitations can be mitigated. The viral dynamic outcomes can then be evaluated depending on the drug exposure profile over time. In this study, we have measured ganciclovir concentrations in real time, which is important as the observed and theoretically expected PK profiles may not always be fully aligned. In further PK/PD modeling analyses, such discrepancies can be appropriately accounted for.

Understanding PK/PD is essential for antiviral agents, as exposure-response parameter relationships are still understudied (15, 29). Particularly for CMV infection treatment, where the therapeutic window is difficult to define as special population groups are impacted (14, 18). This concerns mostly transplant recipients, who are immunocompromised and thus vulnerable to invasive sampling procedures in clinical studies. Our established infection model, thus serves as an advantage when recreating infections in immunocompromised conditions. Especially, in the context of complete absence of immunity, where treatment depends solely on drug efficacy. In the case of ganciclovir, current standard dosing is applied across various patient profiles, which has led to sub-exposure, a slow decline of viral load suppression, and clinical toxicity (8, 16–18, 30). There is, thus, an urgent need to optimize therapeutic targets and evaluate the exposure-effect relationship of ganciclovir for CMV treatment.

Previous studies have used similar HFIM systems to optimize and define therapeutic targets in cases of influenza A, Zika, and HIV infection treatments (3, 31–33). This approach is particulary useful in the light of the recurring emergence of viral resistance, where sufficient drug exposure is critical (34, 35). While the HFIM can offer a human-like drug-exposure profile on a cellular level, current state-of-the-art organ-on-a-chip systems might offer an alternative understanding of PK/PD evaluations on a tissue level and across multi-organ systems (36).

This study has some limitations. The HFIM allows controlled drug concentration exposure, which is beneficial predominantly for studying exposure-response relationships. However, in the case of a nucleoside analog, such as ganciclovir, intracellular drug metabolism might greatly influence the exposure-response relationship (37, 38). Therefore, further setup and analysis for both intracellular drug concentration and viral load measurements need to be investigated during PK/PD analysis. In addition, the role of CMV-specific CD4^+^ and CD8^+^ T cells in controlling CMV infection needs to be considered when translating results into the clinical setting (39, 40). Thus, the interplay between infection and immunity response on the PK/PD outcomes needs to be further evaluated and validated using additional experimental setups. Incorporating these host immune parameters into a viral dynamic model may, however, mitigate these limitations (41).

Finally, this infection model will ultimately support data generated from animal models and clinical trials, thereby paving ways to accelerate PK/PD studies and dose selection. Fundamental understanding of the relationship between ganciclovir exposure and viral dynamics is an essential step for defining clinically relevant targets. Such targets can be evaluated systematically using the HFIM system, which can be applied across multiple stages of drug development in addition to being cost-effective.

In conclusion, we have developed a novel and affordable dynamic *in vitro* model for antiviral treatment optimization studies. The results of our study have shown the feasibility for further PK/PD assessments of ganciclovir in the established HFIM setup. This is especially useful to define and optimize clinical targets of ganciclovir in CMV infection treatment.

## MATERIAL AND METHODS

### Cells and virus

MRC-5, human lung fibroblast cells (ATCC CCL-171) were cultured and maintained at 37˚ C with 5% CO_2_ in Minimum Essential Medium (MEM; Capricorn Scientific) supplemented with 10% fetal bovine serum (FBS; Sigma-Aldrich), 1% GlutaMAX - I CTS (Gibco), 1% non-essential amino acids (NEAA; Gibco), and 1% penicillin-streptomycin (Gibco). Trypsin (Capricorn Scientific) was used for passaging and collection of adherent cells from cell culture flask. Human cytomegalovirus (HCMV) Towne (ATCC VR-977) and the Merlin strains were used for the infection model. The Merlin strain was obtained from BAC-derived clones, which are genetically similar to the Merlin (wild-type) strain (19). This was kindly provided by Professor Richard Stanton, from the Virology Immunology group, Cardiff University. The two strains were propagated in MRC-5 cells, and viral stocks were used for infection. Viral stock titers were quantified by performing the TCID_50_ assay. An MOI of 0.1 (Towne) and 0.01 (Merlin) is used for infection experiments.

### Hollow-fiber infection model

#### Cell culture and virus infection

MRC-5 cells were maintained and expanded in filtered cell culture flasks until confluency is reached, and concentrations were reached for the experiment. Cells (5.0 × 10^7)^ were collected, and the virus was injected altogether into the cartridge through the inoculation ports with syringes. Media containing cells and viruses were mixed carefully from the two ports to allow an even distribution throughout the hollow-fiber cartridge. Thereafter, inoculated cells and virus were incubated at 37˚C with 5% CO_2_ for 3 days to allow settlement and growth of cells and virus.

#### General overview of the HFIM system

A schematic diagram of the HFIM system is presented in Fig. 1. The main circulation loop connects the central reservoir and the dialysis cartridge (FX paed hemodialyzer, Fresenius Medical Care, Bad Homburg, Germany) via a peristaltic pump (DT15, BT100F-1, Lead Fluid). The hollow-fiber is represented by the cartridge that contains semi-permeable polysulfone (Helixone) membranes (fibers) with a pore size of 3.3 nm, separating the intracapillary space (ICS) and the extracapillary space (ECS). This allows diffusion of drug compounds between the ICS and the ECS while keeping the cells and viruses retained in the ECS. The drug is administered with an automatic syringe pump (ALADDIN-1000, World Precision Instruments) toward the central reservoir. To mimic the drug half-life (clearance), a second peristaltic pump was used to add the drug-free cell culture medium to the central reservoir. The third peristaltic pump was set at a slightly higher rate, discarding the excess volume in the central reservoir to the waste. The synergy of the two pumps dilutes the drug while maintaining a constant volume of the medium in the central reservoir. Silicone and polypropylene-based tubes were used to circulate the fluid. Tubes and connections were sterilized beforehand, and the final setup was assembled under the flow hood to maintain sterility.

The flow rate in the main circulation loop was set to 30 mL/min to achieve rapid equilibration of drug concentrations between the central reservoir and the ICS of the cartridge. An automated drug infusion program was used for drug administration where different dosing regimens can be applied: https://github.com/LeidenPharmacology/LeidenSyringePump.

Before the cells and virus were inoculated, the system was primed with DPBS (1×) with Mg^2+^ and Ca^2+^ (Capricorn Scientific) to allow better cell attachment. Afterward, priming was also done with complete growth medium, overnight. Then, the medium containing cells and viruses was introduced via the inoculation port. Collection of virus samples was done via the syringes that connect the two ports of the ECS of the HF cartridge. Drug-containing media samples were collected with syringes through a three-way port between the HF cartridge and the central reservoir.

#### PK parameter estimation and hollow fiber setup reproduce the human plasma PK profile

To reproduce the human plasma PK profile, we targeted 10 mg/L as the peak concentration (C_max_) value, which corresponds to the range observed in patients (42). With the current dosing recommendation at 1-hour continuous infusion (t), the drug infusion rate (K_0_) was calculated based on the following equation (multiple dose):


$$K_{0\;}\left(mg/h\right)=\;\;\frac{CL*Cmax}{\left(1-e^{-K_{e}*t}\right)}$$

For all targeted C_max_ values, the elimination constant was calculated based on a typical half-life (t_1/2_), which was assumed to be 4 h, where


$$K_{e}\left(hour^{-1}\right)*=\frac{ln\left(2\right)\;or\;0.693}{t_{1/2}}$$

Clearance was calculated based on K_e_ and the volume of central reservoir, which was kept constant at 0.2 L,


$$CL\;\left(\frac{L}{h}\right)=\;Volume\;of\;central\;reservoir\;\left(L\right)*K_{e}\left(hour^{-1}\right)$$

Ganciclovir (Thermo Fischer Scientific Chemicals) was freshly prepared at 1 mg/mL in DMSO, and the working solution was prepared and sterile-filtered at 250 mg/L in water. The drug infusion rate (R) was set according to the dose (mg/h), administered every 12 h. The drug infusion rate (R) was calculated according to the following equation:


$$R=\frac{Dose\;\left(mg/h\right)}{Drug\;concentration\;in\;syringe\;\left(working\;solution\;at\;mg/L\right)}$$

### Pharmacokinetic analysis of ganciclovir

Samples collected were stored at −80˚C until ready to be measured by the UHPLC-MS/MS. GCV-D5 was used as an internal standard (Toronto Research Chemicals, Toronto, Canada, G235002). The Shimadzu Nexera X3 UHPLC (Kyoto, Japan) coupled to a SCIEX 6500 + QTRAP mass spectrometer with an electrospray ionization (ESI) source (Sciex, Malborough, MA, USA) was used for bioanalysis. A Kinetex LC Column (Phenomenex, 1.7 µm HILIC 100 Å, 50 × 2.1 mm), integrated with a security guard holder for the column (Phenomenex, 2.1 to 4.6 mm ID), was employed for chromatographic separation. The temperature of the column oven was kept at 30℃. Two sets of mobile phase combinations were used for bioanalysis; 0.1% formic acid and 1 mM ammonium formate served as the aqueous phase (mobile phase A); meanwhile, 99.75% of acetonitrile, 0.1% formic acid, and 1 mM ammonium formate were employed as the organic phase (mobile phase B) to elute ganciclovir. Acetonitrile, methanol, and formic acid were of LC-MS quality (Biosolve BV, Valkenswaard, the Netherlands). Prior to measurement, all samples were spiked with the internal standard and were diluted in methanol and thereafter mixed and centrifuged for 10 min, at 20,000× *g* at 4˚C. The final dilution of supernatants at 1/1,000 was prepared for measurement. A standard curve of ganciclovir was used to quantify samples where 1/x^2^ is equal to or more than 0.99. Three quality control points (0.2, 4, and 40 μg/mL) were used to validate the standard curve. The gradient LC program was started from 100% B for 0.5 min, increased to 50% B and held for 2.5 min, returned to 100% B, and then equilibrated for 2 min. The run time was 5.5 min with a flow rate of 0.3 mL/min. SCIEX OS software platform (version 3.0) was used for acquisition and quantitation processing.

### PK analysis

The observed concentrations sampled from the ICS were computed for fitting estimations. The expected PK profile was simulated using the rxode2 package. The estimation of PK parameters was fitted using a one-compartmental model with the nlmixr/nlmixr2 package in R software (2024.12.1+563).

### DNA extraction and quantitative PCR for viral quantification

Extracellular viral samples from the ECS were treated with a recombinant proteinase K (Thermo Fischer Scientific) at 65 °C for 30 min, followed by heat inactivation at 90°C for 10 min. DNA was precipitated with isopropanol and washed with 75% ethanol. Afterward, DNA pellets were air-dried and resuspended in 30 µL of nuclease-free water. DNA extracts were stored at −20°C. qPCR was performed using primers targeting the UL83 gene in 96-well fast plates (Applied Biosystems) with the following mix per sample: SensiMix SYBR low-ROX (QT625-05, Meridian BioScience), forward primer ([5′−3′]: GGCTTTTACCTCACACGAGCATT) and reverse primer ([3′−5′]: GCAGCCACGGGATCGTACT), NH_4_Cl buffer (10×, GC Biotech), and MgCl_2_. Plates were run using a standard 40-cycle, with the ABI 7500 system. Standard curves were generated from a 10-fold serial dilution of the CMV genomic DNA (VR-538, ATCC).

### Viral titer quantification with TCID_50_

MRC-5 cells were seeded at a cell density of 1.5 × 10^4^ cells/well in 96-well plates, overnight at 37°C with 5% CO_2_. Tenfold serial dilutions of the viral samples were performed in six replicates (20 μL/well). The cytopathic effect (CPE) of each well was evaluated, where positivity is defined when less than 25% of adherent cells were observed. TCID_50_/mL and the predicted value of PFU/mL (conversion factor 0.7) were calculated with a TCID_50_ calculator developed by Marco Binder, University of Heidelberg, based on the Spearman-Kärber method.

### Statistical analysis

Visual plots and statistical analysis were performed using the PRISM10 GraphPad and R Software. To visualize statistical differences, the mean ± SD is reported. To assess the statistical significance between drug concentration samples from the ICS and ECS, we first performed normality tests and then a paired *t*-test. A *P*-value of ≤0.05 was significant.

## SUPPLEMENTAL MATERIAL

Supplemental figures aac.00381-26-s0001.docx Fig. S1 and S2.

ASM does not own the copyrights to Supplemental Material that may be linked to, or accessed through, an article. The authors have granted ASM a non-exclusive, world-wide license to publish the Supplemental Material files. Please contact the corresponding author directly for reuse.
