# Erratum for Sudarsono et al., “A novel hollow-fiber infection model (HFIM) for antiviral PK/PD studies of CMV infection”

**DOI:** 10.1128/aac.01045-26

**Published:** 2026-09-23

**Authors:** Lalitya M. Sudarsono, Suzanne A. M. Wenker, Xuanlin Liu, Jorn Brink, Dirk-Jan van den Berg, J. G. C. van Hasselt, Anne-Grete Märtson

## ERRATUM

Volume 70, no. 8, e00381-26, 2026, https://doi.org/10.1128/aac.00381-26. An incorrect label was used. “AUC_24h_” should read “AUC_7h_” in Fig. 4D and throughout.

