# Supplementary material for: A novel hollow-fiber infection model (HFIM) for antiviral PK/PD studies of CMV infection
Source: Antimicrob Agents Chemother. 2026 Jun 18;70(8):e00381-26. doi: 10.1128/aac.00381-26 (PMC13435646; doi:10.1128/aac.00381-26)
Supplement: Supplementary file 1 [file aac.00381-26-s0001.docx]

**Supplementary figures**

**Figure S1. Log_10_ fold change of CMV replication in MRC-5 cells within the HFIM system.** Circle and square lines represent the Towne (lab-adapted) and the Merlin (wild-type) strain, respectively. (**A, B**) represent the log_10_ fold change relative to quantified copies/mL and PFU/mL at day of inoculation, over 6 days of infection period at 37˚ C/5% CO_2_.

**Figure S2.** **CMV genome and titer production over time, with and without ganciclovir treatment in HFIM.** Ganciclovir treatment was administered 2 mg/hour, 1-hour continous infusion, every 12 hours, 3 days post-inoculation. Circle and square lines represent the control (no drug treatment) and drug treatment group, respectively. (**A-D**) represent the genome copies (copies/mL) and virus titers (PFU/mL) for Towne and Merlin, over 72 hours post-treatment initiation.
